# Field Resistance to Rose Rosette Disease as Determined by Multi-Year Evaluations in Tennessee and Delaware

**DOI:** 10.3390/pathogens12030439

**Published:** 2023-03-10

**Authors:** Mark T. Windham, Thomas Evans, Sara Collins, Juniper A. Lake, Jeekin Lau, Oscar Riera-Lizarazu, David H. Byrne

**Affiliations:** 1 Department of Entomology and Plant Pathology, University of Tennessee, Knoxville, TN 37996, USA; 2 Department of Plant and Soil Science, University of Delaware, Newark, DE 19716, USA; 3 Department of Horticultural Sciences, Texas A&M University, College Station, TX 77843, USA

**Keywords:** rose rosette emaravirus, *Emaravirus rosae*, *Phyllocoptes fructiphilu*, *Rosa*

## Abstract

Rose rosette disease (RRD) caused by the rose rosette emaravirus (RRV) and transmitted by the eriophyid mite *Phyllocoptes fructiphilus (Pf)*, both native to North America, has caused significant damage to roses over the last several decades. As cultural and chemical control of this disease is difficult and expensive, a field trial was established to systematically screen rose germplasm for potential sources of resistance. One hundred and eight rose accessions representing the diversity of rose germplasm were planted in Tennessee and Delaware, managed to encourage disease development, and evaluated for symptom development and viral presence for three years. All major commercial rose cultivars were susceptible to this viral disease to varying levels. The rose accessions with no or few symptoms were species accessions from the sections Cinnamomeae, Carolinae, Bracteatae, and Systylae or hybrids with these. Among these, some were asymptomatic; they displayed no symptoms but were infected by the virus. Their potential depends on their ability to serve as a source of viruses. The next step is to understand the mechanism of resistance and genetic control of the various sources of resistance identified.

## 1. Introduction

Rose rosette disease, caused by the rose rosette emaravirus (*Emaravirus rosae*), was first reported in the western part of North America and has since moved to the eastern coast. Within the last couple of decades, it has emerged as the major disease in cultivated roses in North America [1]. Common symptoms across host cultivars include witches’ broom (shoot proliferation), hyper-thorniness, enhanced red shoot coloration, strapped leaves, and distorted flowers, all leading to poor plant growth and eventual death [1,2,3,4,5]. RRV infection spreads systemically and has been detected in the roots, stems, leaves, and flowers of infected plants [6,7].

RRV has a segmented negative-sense RNA genome enclosed by a double membrane envelope [8,9,10,11]. The genetic diversity among RRV isolates from 16 states revealed little geographic clustering, likely due to the commercial movement of infected plants [9,12]. Nucleotide similarities among examined genomes were from 97.7 to 100% [12].

RRV is dispersed by wind via the eriophyid mite *Phyllocoptes fructiphilus*. This mite primarily resides in protected areas within floral tissues and secondarily in leaf bases, preferentially feeding on tender new growth. It overwinters under the bark, old bud scales, flowers, and other protected places on the plant. *Pf* populations have a short life cycle and are prevalent throughout the growing season with periodic population spikes [13,14,15]. Mites become viruliferous after 5 days of feeding on infected plants but can transmit RRV in less than one hour [6]. Susceptible roses infested by viruliferous *Pf* can develop symptoms after 1 to 6 months [16,17,18]. *Pf* is widely distributed on wild and cultivated roses [19] with southern and northern geographic limits [20].

Rose rosette disease (RRD) is a widespread threat in the USA to rose producers, landscapers, and consumers [1,21,22,23,24,25], having caused the death of hundreds of thousands of roses in private/public gardens, commercial landscapes, and nurseries. RRD is difficult to control due to its dispersal by wind via the eriophyid mite *Pf*, the prevalence of asymptomatic plants in commerce, and the susceptibility of rose varieties. This disease also poses a grave risk to rose cultivation throughout the world if it is spread to major cut-flower and garden rose production zones in Europe, South America, Africa, and Asia [26].

The first reports about resistance to RRD among roses comes from grafting work, which indicated that several North American species (*R. arkansana* Porter, *R. blanda* Aiton, *R. carolina* L., *R. californica* Cham. and Schon., *Rosa palustris* Marsh, and *R. setigera* Michx.) and one Asian species (*R. spinossisima* (L.)) did not develop symptoms of the disease after grafting infected buds onto them and were considered resistant to RRD [19,27]. Two species, *R. arkansana* Porter and *R. woodsii* Lindl., were reported as tolerant to the viral disease. Other species, including three from North America (*R. pisocarpa* Gray, *R. nutkana* Presl., and *R. woodsii var. ultramontana* (Wats)) and 12 with Asian or European origins, appeared to be susceptible to RRD. These Eurasian species included *R. banksiae* Aiton, *R. canina*, *R. dumetorum* Thuill, *R. eglanteria* L., *R. rubrifolia* Vill., *R. soulieana* Crep., *R. spinosissima var. altaica* (L.) Rehd., *R. montezuma* Hum and Bonpl., *R. multiflora* Thunb., *R. odorata* (Andr.) Sweet, *R. gallica* L., *R. hugonis* Hemsl., *R. villosa* L., and *R. wichurana* Crepin. These species belong to eight different sections of the genus *Rosa* and thus represent wide genetic diversity. It should be noted that RRD resistance in one accession of a species cannot be extrapolated to all accessions of the species.

This early work reported that all 51 cultivated accessions of rose examined were susceptible or very susceptible to the disease, although one floribunda rose Bonica (‘MEIdomonac’) was reported to be resistant to the mite but not the pathogen. This included a wide range of diversity, including climbers, hybrid rugosas, floribundas, hybrid teas, miniature, polyanthas, one rootstock, and 14 Old Garden Roses [19]. Subsequent observational data collected from rosarians, state-level extension employees, and researchers report RRD symptoms and disease development in 1000 rose cultivars and 42 rose species accessions. The germplasm evaluated includes a diversity of modern roses (hybrid tea, shrub, floribunda, grandiflora, miniatures, and polyanthas), Old Garden Roses (China, tea, damask, centifolia, gallica, moss, musk, and Portland), and a wide range of hybrid rose species, of which hybrid *multiflora* and hybrid *wichurana* rose accessions are the most common. Although cultivated roses are susceptible to RRD, differences in susceptibility among cultivars were observed.

Work in Arkansas showed that the roses Bonica and Homerun (‘WEKcisbako’) were difficult to infect and that the rose Stormy Weather (‘ORAfantanov’) was not infected by either budding with an infected bud or by the feeding of infected mites [6]. The floribunda Bonica was previously reported to be resistant to the mite but not the pathogen [19].

Recent research identified several genes/quantitative trait loci (QTL) for partial resistance to RRD [28,29] in an interconnected multi-parent diploid population (six F_1_ populations with nine parents) as well as two connected tetraploid populations involving three cultivars (My Girl (‘BAIgirl’), Stormy Weather™, and Brite Eyes™ (‘RADbrite’)). A QTL located on linkage group (LG) 5 coincided in both diploid and tetraploid germplasm, had the greatest effect on RRD resistance (explaining 20 to 41% of the phenotypic variance), and was consistently detected in multiple datasets. The sources of the RRD resistance QTL on LG5 were traced back to the tetraploid variety Brite Eyes™ and the diploid TAMU breeding line M4-4, both with *Rosa wichurana* in their parentage. Additional minor QTLs were identified on LG1, LG3, LG6, and LG7.

Mite reproduction studies on 32 modern rose cultivars have shown that modern roses are good hosts for *Pf* [6,19,30], although additional work with greater genetic diversity of rose cultivars is necessary to assess the resistance of cultivated roses to the mite vector. In contrast, *Pf* was not able to reproduce on two rose species accessions (*R. bracteata* and *R. carolina*) or plants from related genera in the Rosaceae family.

Roses are one of the most popular plants in public gardens and commercial/residential landscapes. Ornamentals, including roses used in home, private, and public landscapes, promote human well-being, enhance air and water quality, reduce runoff and erosion, facilitate rain capture and stormwater management, reduce noise and dust pollution, and increase property values [31]. The rose industry contributed USD 777 million in direct economic impacts to the US economy in 2014 [32]. Rose growers produced ~37 million garden rose bushes worth USD 203 million in 2014 but only ~25 million bushes worth USD 168 million in 2019. There has also been a decrease in shrub rose producers from 1808 to 1469 over this same period [33,34]. These losses are due primarily to the RRD epidemic [1] and black spot disease.

The landscape rose market accounts for 35% of the roses sold, but this share recently decreased by 10–15% due to RRD. The estimated loss of rose sales from the RRD epidemic is USD 5–10 million annually. A recent introduction of RRD into a California production block resulted in expenses of over USD 1,000,000 to eliminate the threat and has led others to change their protocols at a high cost to prevent the disease from getting into their production blocks. Increased production costs drive up rose wholesale and retail prices while depressing rose sales in a market already being devastated by the fear of RRD. This is reflected in a 23% decrease in annual value (adjusted for inflation), a 33% decrease in the number of shrub roses produced, and 19% fewer businesses producing shrub roses from 2014 to 2019 [33,34].

Since there is no chemical control of the virus, current control approaches include exclusion via the use of clean stock material, the decrease in inoculum/vector populations by roguing of symptomatic plants in and around the garden, the use of barriers to slow the vector’s spread, and chemical control of the vector [5]. An approach that is currently lacking is the use of resistant cultivars. Disease resistance is not only the easiest control option to employ but also the most desired trait among consumers and the industry [35,36]. The willingness to pay for this and other adaptation traits is in the range of USD 10 to USD 15 per plant [32]. Producers and breeders benefit from long-market-life cultivars through increased returns for product investment. The economic impact of RRD-resistant rose cultivars is estimated to be at least USD 35 million/year. These improvements will lower the cost of production, increase the ease of growing, and increase consumer satisfaction with garden roses.

The objective of this field trial was to evaluate a suite of roses that represent the wide genetic diversity of the cultivated and wild roses for their resistance to RRD.

## 2. Materials and Methods

### 2.1. Plant Materials

A total of 108 rose accessions including rose species and a wide array of cultivars (Table 1) were planted in field plots near Crossville, TN, at the University of Tennessee (UT) Plateau Research and Education Center (35.96° N, −85.04° E, 565 m) in July 2015 and at the Agricultural Experiment Station of the University of Delaware’s College of Agriculture and Natural Resources in Newark, Delaware (39.68° N, −75.75° E, 40 m), in May and June 2015. Among these roses were hybrid teas (9), shrubs (27), floribundas (20), climbers (5), miniature (4), polyanthas (3), grandifloras (4), noisette (1), bourbon (1), China (2), rootstocks (3), species hybrids (15), and species accessions (14). The most common species hybrids were those with *Rosa rugosa* parentage. Most of the accessions were genotyped using the Axiom WagRhSNP 68K SNP array, and their genetic relationships were assessed using Prevosti’s distance [37], a measure of relative dissimilarity as implemented in the R-package ‘poppr’ [38]. Using Prevosti’s distance, we generated a nearest joining neighbor tree with the R-package ‘ape’ [39] and visualized the tree with the R-package ‘ggtree’ [40].

### 2.2. Field Plot Design

The Tennessee (Lily loam) and the Delaware (Elsinboro silt/loam) sites are in the USDA Hardiness zones 6b and 7a, respectively. The fields were organized using a randomized block design with three replications. Each rose plant was an experimental unit. In Tennessee, roses were planted 2.4 m apart in a staggered double rose configuration that was 1.8 m apart. Roses were mulched ~8 cm deep and irrigated with drip irrigation. Irrigation was run to ensure that the plants received one inch of water per week. Fertilization was conducted according to soil test data received each year from the UT Soil and Plant Disease/Pest Diagnostic Center (Nashville, TN, USA). In Delaware, the rows were spaced 2.4 m apart. Within rows, roses were planted with 0.9 m spacing between each plant. Supplemental watering was provided to the rose, initially by hand and later via drip irrigation. The irrigation was run up to twice a week depending on the rainfall, temperature, and plant age. Roses were planted by hand and kept weeded, mulched, and pruned during the growing season to encourage the growth of soft tissue, which is required by the eriophyid mite vector for feeding and reproduction. No fungicides or insecticides were sprayed during the trials.

### 2.3. RRD Augmentation Procedures

At both locations, there were *Rosa multiflora* plants infected by the virus and infested by the vector mite (*Phyllocoptes fructiphilus*), which served as a natural source of the virus and mite. In Tennessee, Knock Out Roses^®^ infected with rose rosette emaravirus (RRV) and infested with the vector mite were dug from a commercial bed in Nashville, TN, and transplanted into the experimental block to enhance disease pressure. The plants in both locations had disease pressure augmented by the release of large numbers of viruliferous mites (600–1200 mites per plant) directly to the roses being screened. This was performed by collecting rosetted shoots from RRV-infected roses and attaching sprigs of these (5–15 cm in length) by clipping with binders or by twist-tying to actively growing shoot tips of a target rose. Augmentation was performed 2–4 times per season with at least one symptomatic shoot attached to each test rose.

### 2.4. RRD Evaluation in the Field

Plants were evaluated for RRD symptoms several times from July to October each year. In Tennessee, the plants were rated for the severity of symptom development using a 0 to 3 scale (0 = no symptoms, 1 = small single shoot with rosetting, 2 = 2–3 shoots with rosetting, and 3 = 4 or more shoots with rosetting), whereas in Delaware, the plants were rated either with no symptoms or with RRD symptoms. Thus, for analysis, the severity scores were transformed into a binary score for each year and replication. The average score per plant over the 3-year trial was used as the phenotypic score for RRD. Thus, if the plant had RRD symptoms all three years, it had an average score of 1.0, and if the plant was asymptomatic all three years, the average score would be zero.

In both locations, plants were tested for the presence of RRV using the RRV2 primer pair [41]. In Tennessee, all plants were tested, whereas in Delaware, only symptomatic plants were tested for the presence of the virus. Samples of leaf tissue were either processed while fresh or kept frozen at −80 °C until processing. In Delaware, approximately 100 mg of symptomatic leaf tissue from each rose was submerged in liquid nitrogen and agitated in a Mini-Beadbeater for 40 s. Total RNA was extracted using Qiagen’s RNeasy Plant Mini Kit. In Delaware, RT-qPCR amplification was performed on the same day. The products were visualized on a 2% agarose gel. In Tennessee, the viral RNA from 2 gm of homogenized rose tissue was extracted using the antigen capture protocol [42]. Virus detection was performed using RT-qPCR [41]. The cycle threshold (Ct) values for the detection of RRV were between 5 and 37 cycles. Samples with Ct values less than or equal to 29 and greater than 5 were considered strong positives, while samples between 30 and 37 were considered moderate to weakly positive for the presence of RRV [7].

### 2.5. Data Tabulation and Analysis

A mixed model was used to analyze the mean RRD evaluations over years with the rose accession as a fixed variable and location as a random variable using JMP Pro 16 software. The calculated means were compared to the overall mean, and the rose accessions were divided into three groups according to rose rosette symptom development—low, moderate, and high. The separation of the low and high groups from the mean score was performed at the 0.10 significance level. The results of virus testing are overlaid on these scores in Table 2, Table 3 and Table 4.

## 3. Results and Discussion

### 3.1. Disease Development over Years

The spread of the disease throughout the experimental plot as indicated by symptom development was low in the first year (0.08), increased four-fold in the second year to 0.36, and peaked in the third year at 0.47. This general pattern is seen for both locations, although the Delaware location, especially in the first year, had lower levels of symptom development than the Tennessee site. Thus, even with augmentation, it takes a three-year field trial to have good confidence in the plant’s ability to resist the infection by this disease.

### 3.2. Cultivar × Location Interaction

The interaction between the cultivar and location was significant although the variation explained by the cultivar was much higher. Upon inspection of this interaction, it was clear that the Delaware site did not differentiate among the levels of disease incidence as did the Tennessee location. This is reflected in the greater number of cultivars with low symptom development in Delaware (34 roses) as compared to Tennessee (19 roses). All disagreements among cultivars were explained by the movement of cultivars among adjacent categories (i.e., low and moderate or moderate and high). This suggests that the interaction effect is due to non-uniform disease pressure rather than variation in virus pathogenicity.

### 3.3. Genetic Relationships of the Roses

The modern rose is a multi-species complex that was developed initially by combining species from the Gallicanae and Indicae sections, which was followed by using species mainly from the Systylae section and, to a lesser extent, species from the Cinnamomeae and Carolinae sections [43,44,45]. The roses tested represent this diversity of the rose group.

**Figure 1:**
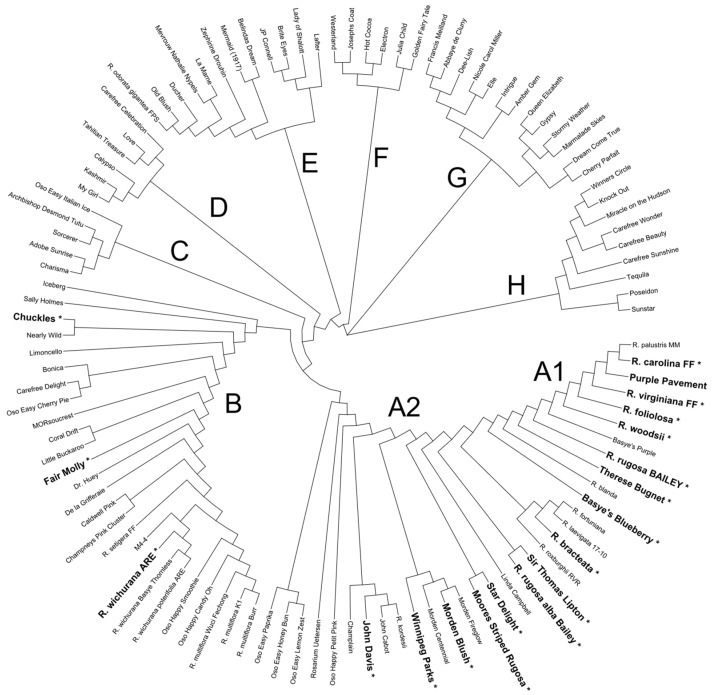
Nearest joining neighbor tree using Prevosti’s distance [37] showing genetic relationships among roses. Data used: Axiom WAgRhSNP array. Names followed by an asterisk (*) and in bold font mark individuals that showed little to no RRD symptoms.

In our genetic relationship tree (Figure 1), the A group includes species within the sections Cinnamomeae, Carolinae, Banksianae, Laevigatae, Platyrhodon, and Bracteatae and rose cultivars with introgressions from species mentioned in the first two sections. The subgroup A1 contains the species within these sections as well as recent hybrids with *R. rugosa* (Purple Pavement, ‘Basye’s Purple’, ‘Therese Bugnet’, ‘Sir Thomas Lipton’, Linda Campbell, Star Delight, and Moore’s Striped Rugosa) and with *R. carolina* and *R. virginiana* (‘Basye’s Blueberry’). The A2 subgroup contains the cultivars with more distant introgressions with these species. As expected, the Canadian cultivars (‘Winnipeg Parks’, ‘Morden Centennial’, ‘Morden Fireglow’, ‘Morden Blush’, ‘John Davis’, ‘John Cabot’, and ‘Champlain’) cluster together within this A2 group. These have parentage derived from *R. kordesii*, *R. arkansana*, and *R. spinosissima*. It is in this A group where most of the roses with no or few symptoms are found.

The B group contains several species of the Systylae section (*R. multifora*, *R. wichurana*, and *R. setigera*) as well as cultivars with recent introgression from these species. Within this group are two roses that showed no or few symptoms. The predominance of floribundas in group C indicates a significant influence of the Systylae species, especially *R. multiflora*. There is a lessening of Systylae background in group D with primarily shrub and grandiflora types. Group E contains species from the Indicae section and related cultivars. The hybrid tea types, which consist of intercrosses primarily among species in the Gallicanae and Indicae sections, are within the F and G groups, along with a few floribundas, grandifloras, and climbing shrubs. Group H, which includes cultivars such as Knock Out^®^, Miracle on the Hudson™, and Carefree Beauty™, are distinct from groups F and G, indicating some introgressions from the section Systylae and perhaps Cinnamomeae. As such, the collection evaluated does represent the wide diversity seen among commercial rose germplasm.

### 3.4. RRD Resistance among Roses

The combined analysis of 108 rose accessions showed a wide range of symptom development, with all of the roses showing no or few symptoms in groups A and B. This ranged from all plants at both sites being asymptomatic to all plants at both sites being symptomatic all three years. There were 21, 67, and 20 accessions that were rated as having low, moderate, and high symptom development (Table 2, Table 3 and Table 4). Most commercial cultivars included in this trial showed moderate to high symptom development.

Among those with no or low symptom development are nine species accessions. Accessions of five North American species (*R. arkansana*, *R. carolina*, *R. folialosa*, *R. virginiana*, and *R. woodsii*) and one Asian species (*R. rugosa*) belong to two closely related sections, Carolinae and Cinnamomeae [46]. Previous work has reported that accessions of several North American species from the subgenus Carolinae (*R. arkansana*, *R. blanda*, *R. carolina*, *R. californica*, and *R. palustris*) did not show symptom development when they were grafted with infected buds [19], indicating that there are strong sources of resistance to RRD among this group of rose species native to North America.

Unfortunately, these have been seldom used in rose breeding due to sexual incompatibility with cultivated roses, with a couple of exceptions. Dr. Robert Basye, in his breeding in Texas, used two species from this group (*R. carolina* and *R. virginiana*) to develop the rose ‘Basye’s Blueberry’ [47], which also showed few to no symptoms in this trial. The species *R. arkansana* and *R. spinosissima*, both of which have been previously reported to be resistant to RRD [19], are in the background of the Canadian roses ‘Morden Blush’, ‘Morden Centennial’, ‘Morden Fireglow’, and ‘Winnipeg Parks’ bred by Henry Marshall. These species were incorporated by crosses with a local accession of *R. arkansana* as well as through his use of the roses ‘Prairie Princess’ and ‘Assinboine’. ‘Morden Blush’ and ‘Winnipeg Parks’ showed few symptoms, whereas ‘Morden Centennial’ and ‘Morden Fireglow’ showed moderate symptom development. RRV was detected in ‘Winnipeg Parks’ and ‘Morden Centennial’ but not in ‘Morden Blush’ and ‘Morden Fireglow’. These have good fertility with most commercial shrub germplasm and may be good sources of RRD resistance. Currently, there is an experiment with populations developed with ‘Morden Blush’ and ‘Morden Fireglow’ to identify the genes involved in their resistance to RRD.

The other two species accessions with no or low symptoms are *R. bracteata* (section Bracteatae) and *R. wichurana* (section Systylae). *Rosa bracteata,* or the McCartney rose, is native to China but, once introduced into the US, became established in the southeastern USA. *Rosa bracteata* has been reported as resistant to the mite that transmits RRD as the mite was not able to reproduce on it [19]. Unfortunately, this species has not been used much by rose breeders due to its sexual incompatibility with most commercial germplasm derived from roses from the subgenera Systylae, Indicae, and Gallicanae. Successful hybrids such as ‘Mermaid’ (W. Paul, 1918) and Muriel (‘MORmurl’) have poor fertility [45].

Resistance from RRD is found within the Systylae section, which has been used extensively in the commercial breeding of roses [43,44,45]. Here, we report that *Rosa wichurana* thornless ARE showed few symptoms, whereas *R. wichurana poterifolia* ARE and *R. soulieana* RM showed moderate to severe symptoms. In addition, the source of the RRD resistance on LG5 appears to be derived from *Rosa wichurana* ‘Basye’s Thornless’ [28,29]. It is important to note that RRD resistance in one accession does not mean all accessions within the species are resistant. This is clear from the results of this trial as well as previous reports of *Rosa wichurana* [19]. Another member of this subgenus that has been reported to be resistant to RRD is *Rosa setigera* [19], which is a North American native within this mainly Asian group.

Another large group of accessions with low symptom development is the hybrids with *R. rugosa* (‘John Davis’, ‘Fuzzy Wuzzy Red’, Moore’s Striped Rugosa (‘MORbeauty’), Purple Pavement (‘HANpur’), ‘Sir Thomas Lipton’, Star Delight (‘MORstar90’), and ‘Therese Bugnet’). These vary extensively in their sexual compatibility depending on how recently the *Rosa rugosa* was used in the parentage. Those that are one or two generations from the initial cross with *Rosa rugosa*, such as ‘Fuzzy Wuzzy’, ‘Purple Pavement’, ‘Sir Thomas Lipton’, ‘Star Delight’, and ‘Therese Bugnet’, have less fertility. Nevertheless, these have some breeding potential in an introgression program to incorporate RRD resistance into a wide diversity of cultivated roses. This is the first report of RRD resistance in this species, although it should be noted that some *R. rugosa* hybrids, such as ‘Basye’s Purple’, ‘Fru Dagmar Hastrup’, and Linda Campbell (‘MORredrug’), showed moderate symptom development.

The final two accessions that developed no or low symptoms of RRD over the 3-year trial were the floribunda rose Chuckles (‘SIMmimi’) and the miniature rose Fairy Moss (MORfairpol’). Both of these clustered in the B group, which also contained the resistant accession of *R. wichurana*, the TAMU RRD resistant breeding line M4-4, and the previously reported resistant species *R. setigera*. Thus, it is possible that these have some Systylae background from which their resistance to RRD is derived.

Of those with no or low symptoms, about half tested positive for the presence of RRV. These include one of the *rugosa* accessions (*Rosa rugosa* Bailey), *R. wichurana* thornless ARE, Fair Molly, four *Rosa rugosa* hybrids (‘John Davis’, Moore’s Striped Rugosa, Star Delight, and ‘Therese Bugnet’)*,* and one Canadian rose (‘Winnipeg Parks’) with *Rosa arkansana/spinosissima* background. The usefulness of these materials depends on how easily the mite can acquire and transmit the virus from these to a susceptible rose. It is possible that mite populations are lower on these roses because they do not form rosettes in which the *Pf* populations proliferate, but this remains to be studied experimentally. Lower mite populations could reduce the spread of the vector if, as some believe, the mite is more likely to balloon off to find another host plant under high population conditions (James W. Amrine, personal communication).

All other commercial roses and species roses included in this trial showed moderate to severe symptoms of RRD, and most tested positive for RRV. Those cultivars showing moderate RRD symptoms without a positive RRV diagnosis were ‘Caldwell Pink’, ‘Lafter’, Manetti, ‘Morden Fireglow’, and Sorcerer (‘SAVasorc’). These roses generally developed rosettes of an RRV infection but not until late in the trial. To ensure high virus titer, rosetted tissue was tested at least twice with different samples. The potential reasons for this anomaly are the following. (1.) The rose leaf tissue contains inhibitory phenolic compounds that disrupt the extraction and/or PCR reaction [41], causing these tests to be false negatives. (2.) The virus titer was below detectable amounts. (3.) The test did not pick up the specific virus variant present in the rose. (4.) The rosetting symptoms observed were caused by a different virus, pathogen, pest, or abiotic stress. At this point, we cannot distinguish between these alternatives. These roses will be replanted and further observed for symptom development and tested for virus with multiple primers and extraction techniques to properly characterize their reaction to RRV.

Previous greenhouse work conducted in Arkansas found that out of the 25 roses examined for their susceptibility using mite and graft inoculations, 24 were susceptible, with 2 of these susceptible roses (Bonica and Homerun) being difficult to infect [6]. Both Bonica and Homerun showed moderate symptom development in the 3-year field trial. As Bonica was previously reported to be resistant to the mite [19], perhaps its moderate symptom development is related to mite behavior or biology on the rose and not its intrinsic resistance to the virus. Only the climbing rose Stormy Weather resisted infection with both techniques. Surprisingly, in the field trial, Stormy Weather showed high development of symptoms and tested positive for the virus. After confirming the identity of the rose in all trials and sequencing the virus in two locations, there was no obvious reason why the results of the two studies differed. More work needs to be conducted to systematically assess the biological diversity of the pathogen/mite vector and the environmental parameters that may affect the expression of host plant resistance to RRD.

### 3.5. Mechanism of Resistance

Although we have identified roses that did not test positive for RRV and/or develop symptoms of RRD under field conditions, it is not known how the plant is able to resist this viral pathogen or its vector or suppress symptom development. It is possible that the lack of symptom development for *Rosa bracteata* and only moderate symptom development in Bonica is due to their reported mite resistance. If this translates to lower mite numbers or less feeding, this could be a mechanism of resistance protecting these accessions.

It has also been reported that the QTL for partial resistance on LG5 affects both symptom development as well as virus titer as measured by Ct values [28,29]. This implies that this resistance QTL affects virus proliferation in the host plant. Unfortunately, the mechanism of resistance is not known at this point and needs to be further investigated by comparing virus and mite reproduction and behavior on a series of resistant and susceptible cultivars.

## 4. Conclusions

As we examine the genetic background of roses with low RRD symptom development in these trials, common parentage includes species within the Cinnamomeae and Carolinae sections. This is consistent with previous work [19] and suggests that more screening needs to be performed within this group. Low symptom development was also seen with some accessions of *Rosa wichurana*. This species, along with *Rosa setigera,* was reported to have resistance to RRD, which would support future screening of the *R. wichurana*- and *R. setigera*-derived climbing and rambling roses. Many of the roses that developed moderate to high symptoms possess a breeding background containing species primarily in the sections Systylae, Indicae, and Gallicanae.

## Figures and Tables

**Table 1:** Roses (code name) assessed in the field for RRD incidence from 2015 to 2018 in Tennessee and Delaware listed by horticultural class.

**Climber** Brite Eyes, RADbrite Winner’s Circle™, RADwin Rosarium Uetersen, KORtersen Stormy Weather, ORAfantanov Westerland, KORwest **Floribunda** Adobe Sunrise, MEIpluvia Bonica, MEIdomonac Charisma, JELroganor Chuckles (1957) Eyeconic Melon Lemonade, SPRomel Hot Cocoa, WEKpaltlez Iceberg, KORbin Intrigue, JACum Joseph’s Coat Julia Child, WEKvossutono Marmalade Skies, MEIlmonblan Nearly Wild Oso Easy Cherry Pie, MEIboulka Oso Easy Italian Ice, CHEwnicebell Poseidon, KORfriedhar Sevillana, MEIgekanu Sunstar, KORsteimm Tequila, MEIpomolo Cherry Parfait, MEIsponge Mevrouw Nathalie Nypels **Hybrid Tea** Abbaye de Cluny, MEIbrinpay Dee-lish, MEIclusif Electron Elle, MEIbderos Francis Meilland, MEItroni Golden Fairy Tale, KORquelda Gypsy (1972) Michelangelo, MEItelov Queen Elizabeth **Grandiflora** Dream Come True, WEKdocpot Love, JACtwin Nicole Carol Miller, MEIskimov Tahitian Treasure, RADtreasure	**Miniature** Little Buckaroo Oso Happy Petit Pink, Zlemariannayoshida Sorcerer, SAVasorc Fair Molly, MORfairpo **Polyantha** Caldwell Pink La Marne Oso Happy Smoothie, Zlecharlie **Shrub** Basye’s Blueberry Belinda’s Dream Carefree Beauty, BUCbi Carefree Celebration, RADral Carefree Delight, MEIpotal Carefree Sunshine, RADsun Carefree Wonder, MEIpitac Champlain Coral Drift, MEIdrifora Desmond Tutu, KORtutu Easy Elegance Calypso, BAIypso Easy Elegance Kashmir, BAImir Easy Elegance My Girl, BAIgirl J. P. Connell Knock Out ^®^, RADrazz Lafter Limoncello, MEIjecycka Miracle on the Hudson Morden Blush Morden Centennial Morden Fireglow Oso Easy Honey Bun, Scrivjean Oso Easy Lemon Zest, CHEwhocan Oso Easy Paprika, CHEmaytime Oso Happy Candy OH!, Zlemartincipr Red Drift, Meigalpio Winnipeg Parks	**Bourbon** Zéphirine Drouhin **China** Ducher Old Blush **Noisette** Champney’s Pink Cluster **Species** *R. arkansana* FF * R. bracteata RM *R. carolina* FF *R. folialosa* ARE *R. odorata* FPS *R. roxburghii* ARE *R. rugosa alba* Bailey *R. rugosa* Bailey *R. soulieana*-RM *R. virginiana* FF *R. wichurana poterifolia* ARE *R. wichurana* thornless ARE R. woodsii RVR R. x fortuniana **Species hybrids** Amber Gem Mermaid John Cabot John Davis Sally Holmes Basye’s Purple Fru Dagmar Hastrup Fuzzy Wuzzy Red Linda Campbell Moore’s Striped Rugosa Purple Pavement Sir Thomas Lipton Star Delight Therese Bugnet MORsoucrest **Rootstocks** De La Grifferaie Dr. Huey Manetti

* Letters after species name indicate their source. ARE = Antique Rose Emporium, Bailey = Baileys Nursery, FF = Forest Farm, FPS = Foundation Plant Services, RM = Ralph Moore, RVR = Rouge Valley Roses.

**Table 2:** Roses with no or low symptom development ^a^ to rose rosette disease as determined by three-year trials in Tennessee and Delaware.

***Rosa arkansana* FF**	**Chuckles**
***Rosa bracteata*-RM**	Fair Molly
***Rosa carolina* FF**	**Fuzzy Wuzzy Red**
***Rosa folialosa*-ARE**	John Davis
***Rosa rugosa alba* Bailey**	Moore’s Striped Rugosa
*Rosa rugosa* Bailey	**Morden Blush**
***Rosa virginiana* FF**	**Purple Pavement**
*Rosa wichurana* thornless ARE	**Sir Thomas Lipton**
***Rosa woodsii* RVR**	Star Delight
Basye’s Blueberry	Therese Bugnet
	Winnipeg Parks

^a^ These individuals had lower symptom scores than the overall mean at the 0.10 level of significance. The mixed model used had genotype as fixed and the location and interaction terms as random effects. Bold font indicates that no virus was detected in the plant.

**Table 3:** Roses with moderate symptom development ^a^ to rose rosette disease as determined by three-year trials in Tennessee and Delaware.

Abbaye de Cluny	Elle	Mevrouw Nathalie Nypels
Adobe Sunrise	Eyeconic Melon Lemonade	Michelangelo
Amber Gem	Francis Meilland	Miracle on the Hudson
Basye’s Purple	Fru Dagmar Hastrup	Morden Centennial
Belinda’s Dream	Golden Fairy Tale	**Morden Fireglow**
Bonica	Gypsy	MORsoucrest
Brite Eyes	Hot Cocoa	Nicole Carol Miller
**Caldwell Pink**	Iceberg	Old Blush
Carefree Celebration	Intrigue	Oso Easy Cherry Pie
Carefree Delight	John Cabot	Oso Easy Lemon Zest
Carefree Sunshine	Joseph’s Coat	Oso Easy Paprika
Carefree Wonder	J.P. Connell	Oso Happy Petit Pink
Champlain	Julia Child	Oso Happy Smoothie
Champney’s Pink Cluster	Knock Out	Poseidon
Charisma	La Marne	Queen Elizabeth
Cherry Parfait	**Lafter**	Red Drift
De La Grifferaie	Limoncello	*Rosa roxburghii* ARE
Dr. Huey	Linda Campbell	*Rosa wichurana poterfolia* ARE
Dream Come True	Little Buckaroo	Rosarium Uetersen
Easy Elegance Kashmir	Love	**Sorcerer**
Easy Elegance My Girl	**Manetti**	Westerland
Electron	Marmalade Skies	Winner’s Circle
	Mermaid	

^a^ These individuals had scores that were not different from the overall mean at the 0.10 level of significance. The mixed model used had genotype as fixed and the location and interaction terms as random effects. Bold font indicates that no virus was detected in the plant.

**Table 4:** Roses with high symptom development ^a^ to rose rosette disease as determined by three-year trials in Tennessee and Delaware.

Carefree Beauty	*Rosa odorata* FPS
Coral Drift	*Rosa soulieana*-RM
Dee-lish	*Rosa x fortuniana*
Desmond Tutu	Sally Holmes
Ducher	Sevillana
Easy Elegance Calypso	Stormy Weather
Nearly Wild	Sunstar
Oso Easy Honey Bun	Tahitian Treasure
Oso Easy Italian Ice	Tequila
Oso Happy Candy OH!	Zephirine Drouhin

^a^ These individuals had scores that were higher than the overall mean at the 0.10 level of significance. The mixed model used had genotype as fixed and the location and interaction terms as random effects.

## Data Availability

The data presented in this study are available on request from the corresponding author.
